# Analysis of pharmaceutical inventory management based on ABC-VEN analysis in Rwanda: a case study of Nyamagabe district

**DOI:** 10.1186/s40545-023-00540-5

**Published:** 2023-02-24

**Authors:** Ephrem Mfizi, François Niragire, Thomas Bizimana, Marie Françoise Mukanyangezi

**Affiliations:** 1 East African Community Regional Centre of Excellence for Vaccines, Immunization, and Health Supply Chain Management, College of Medicine and Health Sciences, University of Rwanda, Kigali, Rwanda; 2 Department of Applied Statistics, College of Business and Economics, University of Rwanda, Kigali, Rwanda; 3 College of Medicine and Health Sciences, University of Rwanda, Kigali, Rwanda

**Keywords:** ABC-VEN analysis, Essential medicines, Budget expenditure, Priority medicines, Rwanda Medical Supply Ltd

## Abstract

**Background:**

Pharmaceuticals account for a large portion of healthcare spending in healthcare organizations. Their effective inventory management is required to match the cost of stocks with the customer demand and avoid shortage of supplies at any health facility level. This study aimed to analyze pharmaceuticals' inventory management using ABC-VEN analysis.

**Methods:**

The study was conducted at Rwanda Medical Supply (RMS) Ltd, Nyamagabe Branch for products distributed to health facilities in Nyamagabe District catchment area from the financial years 2017–2018 to 2019–2020. It consisted of a descriptive retrospective study of 457 items. The latter are generic essential medicines distributed to public health facilities during the study period. Products were arranged according to a descending order of importance, and we performed a breakdown of products according to the Pareto Principle. Following an ABC analysis of distribution data for such drugs billed to healthcare facilities, a VEN analysis was performed to identify high-value vital products that require more attention.

**Results:**

During the ABC analysis, 76 products were classified in group A. These accounted for 19.84% and had a value of 74.91% of the total cost of all products. Group B included 116 products, representing 30.29% with a value of 20% of the total cost, while Group C had 191 products, representing 49.87% with a value of only 5.09% of the total cost. During the VEN analysis, 202 products (44.20%) were classified as vital, 231 (50.54%) as essential, and 24 products (75.26%) as non-vital. The analysis with ABC-VEN resulted in Class I representing 55.80% of all medicines that cost 87.88% of all total cost, Class II representing 40.70% with a total cost of 11.82%, and Class III representing 3.50% with a cost of 0.3%.

**Conclusions:**

This study results show that inventory management of vital and expensive products, such as antibiotics, antihypertensive pharmaceuticals, consumables, and massive solutions would be carefully monitored to prevent a shortage of such products at health facility levels. The ABC-VEN analysis is one of the practical and affordable method to achieve their optimized supply chain.

**Supplementary Information:**

The online version contains supplementary material available at 10.1186/s40545-023-00540-5.

## Background

The third edition of the WHO Drug Situation Report 2011 recommended that countries increase their efforts to measure and monitor the prices and availability of medicines to respond to observed changes in the cost of medicines [1]. These efforts would improve procurement, reduce medicines prices by eliminating tariffs and taxes on pharmaceuticals, and promote high-quality generic medicines. In addition, profit margins should be regulated to avoid high costs [2]. Health commodities account for a large portion of healthcare spending in healthcare organizations [3, 4]. However, with a frequent shortage of medicines, it would not be easy to ensure the best healthcare service delivery [5–8]. Thus, effective inventory management is needed to match the cost of stocks with the demand for medicines [3, 9, 10]. Optimal medicines management helps ensure that essential drugs are available at affordable prices [1]. Reliable data would, therefore, contribute to an uninterrupted healthcare supply chain by planning and estimating the medicines needed and the total charges in a given period [3, 11, 12].

Different inventory management techniques are used for pharmaceutical expenditure analysis [13]. Among inventory control techniques used, we may enumerate ABC, VEN, FSN, HML, SOS, and ABC-VEN [14]. Among those techniques, ABC-VEN analysis is often preferred since it contributes to analyzing the medicines expenditures based on public health value and cost [12, 15]. ABC analysis enables efficient management of pharmaceuticals, especially in hospitals and healthcare supply chain facilities, helps compare the cost of managed items and may optimize services by predicting inventory level, ordering, and purchasing cost. Combined with VEN analysis, ABC gives better results for products with high priority considering the price and public health importance [4, 7, 13]. This analysis provides information on the budget spent on medicines in general, and on a particular group of medicines. It provides useful information to direct policymakers and managers to plan the required funding for medicines and how it would be allocated [7]. ABC-VEN analysis classifies health commodities into three categories, Category I with vital and expensive products and fast-moving products. Category II represents essential and quite expensive products, while category III represents non-essential and less expensive products [2, 15]. ABC-VEN analysis has been conducted in different countries, giving relevant results supporting health supply chain systems. The studies conducted in India, Soudan, Ethiopia, and Kenya showed that medicines in the A categories consume an enormous amount of products, between 70% and 80% of the total [12, 15–18].

In Africa, studies were conducted in South Sudan, Ethiopia, and Kenya on the inventory control and expenditure of medicines in health facilities and the supply chain, which provided information on the classification of drugs according to ABC-VEN and helped to improve medicine supply and resource management [16–18]. Studies done in Rwanda showed the shortage in essential medicine in terms of availability and affordability [5, 6, 19, 20]. Currently, there is limited literature relating the accessibility of medicines to inventory control techniques in the Rwanda health sector. In particular, the status of pharmaceutical inventory management concerning the budget spent on vital, essential, and non-essential pharmaceuticals in Rwanda is currently unknown. This study aimed to assess inventory management of pharmaceutics using ABC-VEN analysis in Rwanda Medical Supply Ltd, Nyamagabe Branch.

## Methods

### Study area

This study was conducted at Rwanda Medical Supply (RMS) Ltd, Nyamagabe branch, Nyamagabe district, Rwanda. Rwanda Medical Supply Ltd is a company owned by the government of Rwanda and is tasked with ensuring the availability of medicines in the country. It was established in 2020 as a merger of the Central Medical Store, the then Medical Procurement and Production Division (MPPD), and thirty District Pharmacies [21]. The study covered the data from the RMS Ltd, Nyamagabe Branch in Nyamagabe district. The latter is one of the thirty administrative districts of Rwanda, located in the Southern province, with a surface area of 1090 km^2^ and more than 341,491 inhabitants [22]. It is located 188 km from Kigali, the Rwanda’s capital.

### Study design and data

We used a retrospective cross-sectional design to analyze the annual medicine expenditures and explore pharmaceutical inventory control management. The analyses concerned data covering the period from 2017 to 2020.

### Study population and sample

This quantitative research targeted all health products distributed to the Nyamagabe administrative district's health facilities. A list of 457 essential medicines was used for this study. It included all articles distributed to health facilities in essential generic medicines.

The management of essential medicines is different from managing products of vertical programs. All medicines managed at Rwanda Medical Supply Ltd, Nyamagabe Branch, in the category of essential generics medicines, were part of the study. However, the vertical programs medicines such as anti-retroviral, anti-malarial, anti-tuberculosis, and maternal and child health (MCH) commodities were not part of the study. The products of the vertical programs were excluded, since there is a line for good management. Different stakeholders involved in managing vertical program medicines contribute to daily follow-up, quantification, and fund mobilization to mitigate any problem.

### Data collection

A data collection tool was designed to capture information about medicines distributed, quantity, unit price, and total prices of items that are part of the study. The distribution data were collected on essential generic medicines, sales, unit cost, and total cost for a 3-year period spanning from July 2017 to June 2020. The secondary data were collected in the RMS Nyamagabe Branch database and other management support tools: stock cards and reports.

### Data processing and data analysis

Microsoft Excel 2013 was used for data entry and analysis. We used descriptive statistics, including tabular, and numerical methods, to describe the main characteristics. In performing ABC analysis, the total expenditures for each item were calculated and arranged in descending order. Then, we calculated the percentage of cost and the cumulative cost. After that, we classified products into ABC categories. VEN analysis was done using judgmental methods by organizing products using existing references [3, 4, 18].

We conducted an ABC analysis to classify medicines based on their budget expenditure and a VEN analysis to classify pharmaceuticals based on their public health value (Additional file 1: Table S1). After combining ABC and VEN analysis by cross-tabulation, we obtain the ABC-VEN matrix, which is used to form various categories [3, 4, 23].

According to the ABC-VEN analysis (Additional file 2: Table S2), there are nine groups from which three classes of medicines were obtained as follows [23]. Category I contains all expensive or vital products: AV, BV, CV, AE, AN. Category II contains other essential or B group medicines: BE, BN, and CE. Category III includes the cheapest and non-essential medicines: CN.

## Results

### Medicines expenditure according to ABC analysis

The ABC analysis considered the total number of 457 essential generic drugs (Additional file 1: Table S1). It was found that 90 products, which accounted for 19.7% of all products sold, had a value of 77.70% of the total cost of all products, and these products were classified into group A. In Group B, 119 products, which represented 26.03% of all products, were ranked with 17.46% of the total cost, while in Group C, 248 products, which represented 54.27% of all products, were classified with a total cost of only 4.84%.

### Top twenty pharmaceutical products ranked according to the cost during 3 years

Products that take up a large part of the budget include antibiotics, medical consumables such as examination gloves, massive solutions, and medicines to treat non-communicable diseases. All antibiotics in the top twenty commodities represent 15.06% of the total cost of all items. In all forms combined, Amoxycillin represented 9.68% of the total cost of all items distributed at the study site (Table 1).

**Table 1:** List of top twenty products ranked by cost in 3 years

No	Item description	Quantity	Unit cost	Total cost Rwf	%
1	Amoxycillin 500 mg capsules	2,955,800	22	66,069,566	5.05
2	Examination gloves T 7.5	44,047	1484	65,387,250	5.00
3	Hydrophile gauze roll 91 m × 90 cm	5,950	9966	59,299,489	4.54
4	Amoxicillin 250 mg capsule	2,896,000	11	31,558,633	2.41
5	Diclofenac suppository 100 mg	499,695	55	27,650,627	2.11
6	Butylscopolamine 10 mg tablet	569,800	46	26,480,531	2.03
7	Nystatin 500,000 UI TAB	825,700	32	26,410,107	2.02
8	Cloxacillin 250 mg gel	1,738,000	14	25,160,126	1.92
9	Penicillin V 250 mg tablet	1,814,000	13	23,272,419	1.78
10	Sodium chloride 0.9% fl 500 ml	53,651	420	22,509,941	1.72
11	Cromoglycate disodic ophthalmic solution 2%	28,256	753	21,290,893	1.63
12	Amoxicillin 125 mg/5 ml suspension 100 ml	61,065	323	19,743,355	1.51
13	Salbutamol spray 200 doses	12,976	1395	18,107,775	1.38
14	Campher 10% ointment 50 g	43,749	388	16,956,785	1.30
15	Erythromycin 250 mg tablet	779,000	22	16,840,748	1.29
16	Omeprazole 20 mg capsules	1,589,300	10	15,893,684	1.22
17	Ibuprofen 200 mg tablet	3,165,000	5	15,637,688	1.20
18	Paracetamol 500 mg tablet	3,723,000	4	15,137,612	1.16
19	Nifedipine 20 mg tablet	1,427,900	11	15,080,827	1.15
20	Ibuprofen 400 mg tablet	1,466,000	10	14,472,425	1.11

### VEN analysis

In the VEN analysis, 202 products (44.20%) were classified as essential products, 231 products (50.54%) as essential products, and 24 products (5.26%) as non-essential products (Additional file 1: Table S1). Vital products accounted for 38.2% of the total cost, essential products 59.17%, and non-essential products 2.63%.

### ABC-VEN analysis

There were three classes in the ABC-VEN analysis classification: Class I, Class II, and Class III.

According to the ABC-VEN classification in Table 2, Class I accounted for 55.80% of all medicines, corresponding to 87.88% of the total costs. Class II accounts for 40.70% of all medicines, which is 11.82% of the total cost, and Class III accounts for 3.50% of all medicines, which is 0.30% of the total cost.

**Table 2:** Results of ABC-VEN analysis in three main classes

Category	Number of items	Percentage of items	Amount Rwf	Percentage of value
I	255	55.80	1,148,486,802	87.88
II	186	40.70	154,512,583	11.82
III	16	3.50	3,944,756	0.30
Total	457	100	1,306,944,142	100

## Discussion

The objective of this study was to analyze inventory management of health commodities based on ABC-VEN analysis. In fact, conducting ABC or VEN analysis alone has its limitations. ABC analysis ignores the criticality of medicines, and VEN analysis alone also ignores the cost value of pharmaceuticals [14]. Therefore, to take advantage of the complementary benefits of the two methods, ABC-VEN matrix analysis is required to identify products that need strict control [4, 14].

The ABC analysis in this study is comparable to other studies, such as Nang Nwe in Bangkok, Mohamed in Soudan, Deressa, and Nguyen [4, 13, 18, 24]. Nang found that Category A represented 12% of the ABC analysis, with 78% of the total budget. Category B accounted for 22%, with 16% of expenditure, and Category C accounted for 66% and 6% of the total expenditure. Only a few commodities consume significant funds in this study, and only one item accounts for 23% [24]. A survey conducted by Vijaya in India in 2017 found that 23.7% of 414 items belonged to Class A and accounted for 70.5% of the total annual expenditure in selected hospitals in Tamil Nadu, India [13, 25]. Compared to a study by Mohamed in the Republic of Sudan in 2016, the ABC analysis showed that a small number of items representing 16.98% of Class A accounted for 70.19%, while 61.15% of Class C accounted for only 9.92% of the total fund [26]. The similarity of these studies to the present study may be related to the fact that the classification of the ABC analysis follows the category and intervals recommended by Pareto. According to Jobira, in the study done in Ethiopia in 2021, the ABC analysis showed that 12.1% of medicines were assigned to Class A, 10.8% to Class B, and 77.7% to Class C. These classes accounted for 80.1%, 10.8%, and 9.1% of total annual medicine expenditures, respectively. Only 10 (1.8%) accounted for 39.8% of the yearly consumption value in class A products.

Pharmaceuticals that account for a significant portion of the budget include antibiotics, massive solutions, medical consumables, and antihypertensives. The results of this study are related to other studies conducted in different areas. The study conducted in the Republic of Soudan to analyze the medicines procured by the National Medicines Supply Fund (NMSF) for 2015–2017, which included 584 imported items, reached very similar results to our study. In that study, 14% of medicines in category A consumed 75% of the budget, 17% of items in the B category cost 15%, and 69% in category C accounted for 10% of total medicine spending. Among Class A products, sodium chloride solution 0.9% w/v 500 ml solution for intravenous infusion ranks first with a 5% share of total expenditure [18]. This product was among the top twenty products with high cost, ranked fifth with 2.03% of the total cost of items distributed. According to Bochkarev, antibiotics and antihypertensive are among the products that consume a large portion of the pharmaceutical budget [27]. A study conducted in Greek also revealed that antibacterial products consume a large part of the total pharmaceutical expenditure within Hospitals [28]. Our study shows that antibiotics are among the products that consume a large budget, and these results are similar to the survey conducted in South Africa. The study in South Africa by Sharma showed that a large portion of the pharmaceutical expenditure was spent on antibiotics. Approximately 7% of the total medicine price was spent on antibiotics [29]. The first item that consumed a considerable budget in our study, amoxicillin, was among the most commonly procured antibiotics in South Africa [29]. The three medicines accounted for 23.6% of annual medicines expenditures among the most frequently used products. The concerned products are Amoxicillin 500 mg capsules, cloxacillin 500 mg capsules, and ceftriaxone 1gm injections [17].

In VEN analysis, category N represents a small number of products and justifies using standards treatment documents and selection following a national essential medicines list. A study conducted in Sudan showed that 45% of all items were in Class N. The VEN analysis also showed that a small number of items (2.34%) in class V accounted for 5.46%, while class N comprised 45.01% of articles and 26.43% of total funds. The medicine class with the highest expenditure was that of general anti-infectives for systemic use (40.37%), which also contributed the most to the increase in total medicine expenditure (48.59%) [26]. According to Jobira, the VEN classification revealed that 16.9% classified as V and consumed 35.1%, 67.9% grouped as class E, which used 61.3%, 15.2% grouped as N class consumed 3.6% of the total cost [17]. The finding is in line with the Tumaini HL study conducted in Tanzania, which stated that 17% of items were vital, 68.5% items were essential, and 14.5% were non-essential medicines of total procured drugs.

In a study conducted by Jobira in Ethiopia in 2021, the ABC-VEN analysis showed that 26.6% of all items fell into category I, with the most significant proportion in categories A and V, accounting for 84.7% of annual income medicine expenditure. 49.2% and 24.2% of medicines fell into categories II and III, which accounted for only 13.2% and 2.1% of total medicine spending, respectively [17]. The ABC-VEN matrix analysis of the medicines inventory conducted by Woldeyohanins 2020 in Ethiopia revealed that 147 items (66.5%) identified as Category I consume 88.99% of the annual medicine expenditure. The remaining 51 (23.07%) and 23 (10.47%) items belong to categories II and III and consume 9.3% and 1.81%, respectively [30]. The results of the study are similar to those of other studies. According to Sabah, Category I accounted for 16.67% of the medicine population studied, and its expenditure accounted for 71.47% of total expenditure. Category II accounted for 58.69%, and their annual consumption accounted for 27.67% of the total expenditure. The third category, 24.64%, accounted for 0.86% of total expenditure [11]. Products in the first category include antibiotics, anti-inflammatories, antipyretics, antihypertensives, massive solutions, gastroenteritis remedies, medical consumables, medicines for mental health problems, and antacids. One product, camphor ointment, in the first category is non-essential. Omeprazole was ranked among the top medicines with high expenditure, possibly resulting in prescription habits. A study conducted in Tanzania at Mwananyamala regional hospital found the same results while investigating whether proton pump inhibitors, including omeprazole, are among products with high expenditure [10].

It is important to indicate that vertically managed program items were not included in the analysis as the related information is normally not available at a decentralized level.

## Conclusion

Pharmaceuticals are lifesaving, costly, and may be harmful when they are not adequately managed and used. Considering the role of medicines in healthcare delivery, it is crucial to maintain an adequate stock level to ensure an uninterrupted supply chain. The stock level should be determined based on priority and available resources.

An ABC-VEN analysis revealed that the most used medicines, which occupy a considerable budget at the RMS Ltd., Nyamagabe Branch include antibiotics, massive solutions, and antihypertensive commodities. The findings suggest that high importance should be attached to these commodities, laboratory reagents, and vital products. Furthermore, a regular ABC-VEN analysis of medical supplies use is important to update the local and national essential medicines list and categories of health commodities.

## Supplementary Information

**Additional file 1: Table S1.** List of 457 items classified into ABC and VEN.

**Additional file 2: Table S2.** ABC-VEN analysis in subcategories.

## Data Availability

The data sets generated and analysed during the current study are included in the manuscript and are available from the corresponding author on reasonable request.

## Abbreviations

ABC: Always better control
VEN: Vital, Essential, Non-essential materials
FSN: Fast-moving, slow-moving, stationary materials
SDE: Limited in supply, difficult to supply, easy to supply
HML: Materials with high, medium, or low stock value
SOS: Classified as seasonal and non-seasonal materials
RMS Ltd: Rwanda Medical Supply Ltd
