# Supplementary material for: Analysis of pharmaceutical inventory management based on ABC-VEN analysis in Rwanda: a case study of Nyamagabe district
Source: J Pharm Policy Pract. 2023 Feb 24;16:30. doi: 10.1186/s40545-023-00540-5 (PMC10129016; doi:10.1186/s40545-023-00540-5)
Supplement: Supplementary file 1 — Additional file 1: Table S1. List of 457 items classified into ABC and VEN. [file 40545_2023_540_MOESM1_ESM.docx]

**Supplementary information**

S1 Table: List of 457 items classified into ABC and VEN

| **No** | **Item description** | **ABC** | **VEN** | **Quantity** | **Unit Price** | **COST / RWF** | **Percentage** | **Cumulative percentage** |
| --- | --- | --- | --- | --- | --- | --- | --- | --- |
| 1 | Amoxycillin 500mg capsules | A | E | 2,955,800 | 22 | 66,069,566 | 5.06 | 5.06 |
| 2 | Examination gloves T 7.5 | A | V | 44,047 | 1,484 | 65,387,250 | 5.00 | 10.06 |
| 3 | Gauz Roll 90cmx91m, 1.5 Kg | A | V | 5,950 | 9,966 | 59,299,489 | 4.54 | 14.60 |
| 4 | Amoxicillin 250mg capsules | A | E | 2,896,000 | 11 | 31,558,633 | 2.41 | 17.01 |
| 5 | Diclofenac suppo 100mg | A | E | 499,695 | 55 | 27,650,627 | 2.12 | 19.13 |
| 6 | Butylscopolamine 10mg tab | A | E | 569,800 | 46 | 26,480,531 | 2.03 | 21.15 |
| 7 | Nystatin 500000 UI tab | A | E | 825,700 | 32 | 26,410,107 | 2.02 | 23.17 |
| 8 | Cloxacilline 250mg capsules | A | E | 1,738,000 | 14 | 25,160,126 | 1.93 | 25.10 |
| 9 | Penicillin V 250mg tab | A | E | 1,814,000 | 13 | 23,272,419 | 1.78 | 26.88 |
| 10 | Sodium chloride 0,9% fl 500ml | A | V | 53,651 | 420 | 22,509,941 | 1.72 | 28.60 |
| 11 | Cromoglycate sodium eye drop | A | E | 28,256 | 753 | 21,290,893 | 1.63 | 30.23 |
| 12 | Amoxycillin 125mg/5ml syrup 100ml | A | E | 61,065 | 323 | 19,743,355 | 1.51 | 31.74 |
| 13 | Salbutamol spray 0.200mg | A | E | 12,976 | 1,395 | 18,107,775 | 1.39 | 33.13 |
| 14 | Campher Ointment 50g, tube | A | N | 43,749 | 388 | 16,956,785 | 1.30 | 34.42 |
| 15 | Erythromycin 250mg tab | A | E | 779,000 | 22 | 16,840,748 | 1.29 | 35.71 |
| 16 | Omeprazole 20 mg tab | A | E | 1,589,300 | 10 | 15,893,684 | 1.22 | 36.93 |
| 17 | Ibuprofen 200mg tab | A | E | 3,165,000 | 5 | 15,637,688 | 1.20 | 38.12 |
| 18 | Paracetamol 500mg tab | A | E | 3,723,000 | 4 | 15,137,612 | 1.16 | 39.28 |
| 19 | Nifedipin 20mg retard cp | A | E | 1,427,900 | 11 | 15,080,827 | 1.15 | 40.44 |
| 20 | Ibuprofen 400mg cp | A | E | 1,466,000 | 10 | 14,472,425 | 1.11 | 41.54 |
| 21 | Thiamine chlorhydrate 100mg Tab (Vit B1) | A | E | 1,672,000 | 9 | 14,384,290 | 1.10 | 42.64 |
| 22 | Metronidazol 250mg tab | A | E | 3,118,000 | 5 | 14,231,309 | 1.09 | 43.73 |
| 23 | Aluminium Hydroxyde Sirop | A | E | 55,075 | 238 | 13,093,500 | 1.00 | 44.74 |
| 24 | Ringer lactate flacon de 500ml | A | V | 29,434 | 431 | 12,685,237 | 0.97 | 45.71 |
| 25 | Amoxy+ clavulanic acid 625 cp | A | E | 103,122 | 110 | 11,292,137 | 0.86 | 46.57 |
| 26 | Lidocaine 2% +epineph 1.8ml dent inj | A | V | 42,650 | 263 | 11,207,146 | 0.86 | 47.43 |
| 27 | RANITIDINE 150MG tab | A | E | 27,300 | 406 | 11,072,040 | 0.85 | 48.27 |
| 28 | Microscope slides 1 pce | A | V | 159,968 | 63 | 10,138,500 | 0.78 | 49.05 |
| 29 | Minigrip sacs, pce | A | E | 2,077,600 | 5 | 9,970,336 | 0.76 | 49.81 |
| 30 | Surgical gloves T 7.5 B/50 Pairs | A | V | 1,884 | 5,218 | 9,830,873 | 0.75 | 50.57 |
| 31 | POLYGLACTIN 910 USP1, 1/2C 40MM | A | V | 1,084 | 9,038 | 9,796,800 | 0.75 | 51.32 |
| 32 | Paracetamol 120/5ml sp 100ml | A | E | 40,352 | 242 | 9,772,473 | 0.75 | 52.06 |
| 33 | Ciprofloxacine 500mg cp | A | E | 429,700 | 22 | 9,517,400 | 0.73 | 52.79 |
| 34 | Barrier Masks | A | V | 20,000 | 460 | 9,200,000 | 0.70 | 53.50 |
| 35 | Cotrimoxazole à 480mg cp | A | E | 1,055,000 | 9 | 8,971,130 | 0.69 | 54.18 |
| 36 | H.PYLORI 50tests | A | E | 315 | 27,632 | 8,704,160 | 0.67 | 54.85 |
| 37 | Valproate de sodium 300mg cp(depakine) | A | E | 149,700 | 58 | 8,662,580 | 0.66 | 55.51 |
| 38 | Iodine Polyvidone 10%, 200ml | A | V | 7,470 | 1,157 | 8,642,790 | 0.66 | 56.17 |
| 39 | Poch Pack 65x 9.0L+185ML | A | V | 57 | 143,095 | 8,156,400 | 0.62 | 56.80 |
| 40 | Captopril 25mg cp | A | E | 906,500 | 9 | 8,049,229 | 0.62 | 57.41 |
| 41 | Film radiologie 35cm x 35cm,pce | A | V | 1,101 | 7,288 | 8,024,121 | 0.61 | 58.03 |
| 42 | Insuline humaine 100UI/ml lente, inj | A | V | 2,374 | 3,279 | 7,785,192 | 0.60 | 58.62 |
| 43 | Valproate de sodium 500mg cp | A | E | 69,700 | 109 | 7,618,934 | 0.58 | 59.20 |
| 44 | Seringue de 10ml + aiguille 21 G plastic uu | A | V | 176,983 | 43 | 7,564,465 | 0.58 | 59.78 |
| 45 | Phenobarbital 100 mg cp | A | E | 290,100 | 26 | 7,511,086 | 0.57 | 60.36 |
| 46 | Sparadrap à oxyde de zinc 10CM X5M,pce | A | V | 8,222 | 888 | 7,297,367 | 0.56 | 60.92 |
| 47 | Vaccin anti rabique | A | V | 1,220 | 5,777 | 7,048,041 | 0.54 | 61.46 |
| 48 | Nystatine sp fl de 30ml | A | E | 16,983 | 409 | 6,942,292 | 0.53 | 61.99 |
| 49 | Fer sulf 200mg+ Acide folic 0.25mg cp | A | E | 2,891,000 | 2 | 6,641,451 | 0.51 | 62.49 |
| 50 | Beclomethasone spray 250mg | A | E | 900 | 7,313 | 6,581,600 | 0.50 | 63.00 |
| 51 | Glucometer test strip code free B/50 | A | E | 604 | 10,523 | 6,355,835 | 0.49 | 63.48 |
| 52 | Rubella Test IgM/G,1pc | A | V | 6,640 | 954 | 6,336,900 | 0.48 | 63.97 |
| 53 | Insuline humaine 100UI/ml rapide, inj | A | V | 1,807 | 3,391 | 6,127,209 | 0.47 | 64.44 |
| 54 | Alcool denature 96% llitter | A | V | 1,762 | 3,289 | 5,794,697 | 0.44 | 64.88 |
| 55 | Glucosé 5% fl de 500ml | A | V | 10,641 | 538 | 5,722,636 | 0.44 | 65.32 |
| 56 | Methyldopa 250mg cp (abdomen) | A | E | 197,501 | 29 | 5,699,024 | 0.44 | 65.76 |
| 57 | Ceftriaxone 1g powder for injection | A | V | 23,735 | 237 | 5,632,912 | 0.43 | 66.19 |
| 58 | Eau pour Injection 10 ml | A | V | 157,422 | 36 | 5,617,304 | 0.43 | 66.62 |
| 59 | Indomethacin suppo 100mg | A | E | 116,800 | 47 | 5,504,220 | 0.42 | 67.04 |
| 60 | Benzyl benzoate 25% fl 1l | A | E | 808 | 6,711 | 5,422,380 | 0.41 | 67.45 |
| 61 | Acide ascorbique 500mg cp | A | E | 601,000 | 9 | 5,309,024 | 0.41 | 67.86 |
| 62 | Ampicilline inj 1g flacon | A | V | 42,035 | 123 | 5,155,733 | 0.39 | 68.25 |
| 63 | Erytromycine 125mg/ 5ml susp. fl de 100ml | A | E | 10,734 | 475 | 5,100,777 | 0.39 | 68.64 |
| 64 | Cefotaxime 1g pdre inj | A | V | 17,100 | 291 | 4,973,000 | 0.38 | 69.02 |
| 65 | Valproate de sodium 200 mg cp | A | E | 75,512 | 65 | 4,912,484 | 0.38 | 69.40 |
| 66 | CELL PACK 20 L | A | V | 47 | 103,753 | 4,876,400 | 0.37 | 69.77 |
| 67 | Ibuprofen syrup 100ml | A | E | 16,879 | 288 | 4,866,898 | 0.37 | 70.15 |
| 68 | HBsAg 50 tests | A | V | 10,503 | 459 | 4,818,999 | 0.37 | 70.51 |
| 69 | Tinidazole tab 500mg | A | E | 328,100 | 14 | 4,747,173 | 0.36 | 70.88 |
| 70 | Carbamazepine 200mg (tegretol) | A | E | 337,300 | 14 | 4,734,430 | 0.36 | 71.24 |
| 71 | Film radiologie 24cm X 30cm ,pce | A | V | 1,002 | 4,707 | 4,716,080 | 0.36 | 71.60 |
| 72 | TOXO RAPID TEST IgG/lgm 40Test | A | V | 206 | 22,860 | 4,709,120 | 0.36 | 71.96 |
| 73 | Salbutamol 500MicroG/ml amp. Inj | A | V | 12,380 | 366 | 4,535,961 | 0.35 | 72.31 |
| 74 | Tétracycline 1% opht.tube de 5g | A | E | 33,416 | 136 | 4,532,870 | 0.35 | 72.65 |
| 75 | Seringue de 5ml + aiguille 21 G plastic uu | A | V | 163,600 | 28 | 4,512,364 | 0.35 | 73.00 |
| 76 | Prednisolone 5mg cp | A | E | 647,000 | 7 | 4,479,054 | 0.34 | 73.34 |
| 77 | Paracetamol 250mg Suppo | A | E | 81,410 | 54 | 4,391,444 | 0.34 | 73.68 |
| 78 | Gant gynecologique sterile | A | V | 7,550 | 578 | 4,363,924 | 0.33 | 74.01 |
| 79 | Hydrochlorothiazide 25mg cp | A | E | 862,000 | 5 | 4,245,945 | 0.32 | 74.34 |
| 80 | Vitamine K inj | A | V | 15,971 | 258 | 4,118,764 | 0.32 | 74.65 |
| 81 | Chlorpheniramine 2mg sp 60ml | A | E | 24,094 | 171 | 4,110,623 | 0.31 | 74.97 |
| 82 | Métronidazole sirop 125mg/5ml fl de 100ml | A | E | 14,387 | 285 | 4,093,649 | 0.31 | 75.28 |
| 83 | Cimetidine 400 mg cp | A | E | 336,000 | 12 | 4,024,017 | 0.31 | 75.59 |
| 84 | Doxycycline 100mg cp | A | E | 311,000 | 13 | 3,995,061 | 0.31 | 75.89 |
| 85 | Cotrimoxazole à 240mg /5ml sp fI de100ml | A | E | 15,122 | 264 | 3,985,401 | 0.30 | 76.20 |
| 86 | Microcuvettes hb 201 B/200 | A | V | 20 | 199,167 | 3,983,333 | 0.30 | 76.50 |
| 87 | Catheter court IV n°24 pce | A | V | 37,459 | 106 | 3,980,503 | 0.30 | 76.81 |
| 88 | Catgut chrome dec 3.5(2/0),PR 1/2C, 40MM | A | V | 1,284 | 3,086 | 3,962,995 | 0.30 | 77.11 |
| 89 | HCV 40 Test | A | V | 394 | 9,938 | 3,915,480 | 0.30 | 77.41 |
| 90 | Set de perfusion +aiguille pce | A | V | 33,448 | 114 | 3,807,405 | 0.29 | 77.70 |
| 91 | URINE TEST STRIP | B | V | 499 | 7,312 | 3,648,585 | 0.28 | 77.98 |
| 92 | Indomethacin gelules 25mg cp | B | E | 953,000 | 4 | 3,509,380 | 0.27 | 78.25 |
| 93 | Cloxacilline sp 125mg/5ml 100ml | B | E | 10,564 | 331 | 3,496,266 | 0.27 | 78.52 |
| 94 | Chlorhexidine 1,5 %+Cétrimide 15 % 1l | B | E | 1,282 | 2,721 | 3,488,138 | 0.27 | 78.78 |
| 95 | CRP Latex And COntrol 100 Test | B | V | 304 | 11,464 | 3,484,913 | 0.27 | 79.05 |
| 96 | Misoprostol (Cytotec) 0.2mg cp | B | V | 9,108 | 377 | 3,438,246 | 0.26 | 79.31 |
| 97 | Vaseline Blanche Pot de 1/2Kg | B | E | 1,567 | 2,191 | 3,433,020 | 0.26 | 79.58 |
| 98 | Cloxacilline 500mg inj | B | V | 24,000 | 139 | 3,337,469 | 0.26 | 79.83 |
| 99 | Solution physiologique gttes nasales 0.9% | B | N | 9,409 | 353 | 3,323,032 | 0.25 | 80.09 |
| 100 | Multivitamines sirop fl de 100ml | B | E | 12,945 | 256 | 3,315,756 | 0.25 | 80.34 |
| 101 | Diclofenac 50mg cp | B | E | 1,275,000 | 3 | 3,310,135 | 0.25 | 80.59 |
| 102 | Masque nez + Bouche (Facial) | B | E | 36,724 | 90 | 3,299,460 | 0.25 | 80.85 |
| 103 | Griséofulvine 500mg cp | B | E | 66,700 | 48 | 3,175,939 | 0.24 | 81.09 |
| 104 | Calcium gluconate Inj 10ml | B | V | 7,465 | 420 | 3,138,164 | 0.24 | 81.33 |
| 105 | Acétylsalicylique acide 500mg cp | B | E | 735,000 | 4 | 3,133,992 | 0.24 | 81.57 |
| 106 | Specimen container pr Selle | B | E | 39,103 | 80 | 3,119,804 | 0.24 | 81.81 |
| 107 | Paracetamol suppo 125 mg | B | E | 61,405 | 50 | 3,094,058 | 0.24 | 82.04 |
| 108 | S.R.O sachet 20.5 g pour 1l | B | E | 47,111 | 64 | 2,998,403 | 0.23 | 82.27 |
| 109 | Métronidazole 500mg 100ml inj | B | V | 10,900 | 272 | 2,965,980 | 0.23 | 82.50 |
| 110 | Stromatolyser 4 DL 2L | B | V | 32 | 91,387 | 2,924,368 | 0.22 | 82.72 |
| 111 | Bande de crêpe 10cm*4m 1 pce | B | V | 13,315 | 213 | 2,838,006 | 0.22 | 82.94 |
| 112 | Mebendazole sp 100mg/5ml fl de 30ml | B | E | 14,801 | 191 | 2,828,368 | 0.22 | 83.16 |
| 113 | Ampicilline 500 mg pdre inj, | B | V | 31,885 | 88 | 2,800,460 | 0.21 | 83.37 |
| 114 | Specimen container pr urine fl | B | V | 32,200 | 86 | 2,756,000 | 0.21 | 83.58 |
| 115 | Oxytocine inj. 10UI, IV | B | V | 31,325 | 87 | 2,733,024 | 0.21 | 83.79 |
| 116 | Amoxy+ acide clavilanique 100mg/12.5mg/ml | B | E | 1,367 | 1,978 | 2,703,359 | 0.21 | 84.00 |
| 117 | Bétamethasone 0.1% 15gr creme | B | N | 12,517 | 214 | 2,682,390 | 0.21 | 84.20 |
| 118 | RPR 150tests | B | V | 300 | 8,868 | 2,660,341 | 0.20 | 84.41 |
| 119 | Levomepromazine 25mg cp | B | E | 13,900 | 188 | 2,619,758 | 0.20 | 84.61 |
| 120 | Catheter Court IV, UU. G 18 | B | V | 24,215 | 105 | 2,532,960 | 0.19 | 84.80 |
| 121 | Spironolactone 25mg, cp | B | E | 61,100 | 41 | 2,499,898 | 0.19 | 84.99 |
| 122 | Coton hydrophile rl 500g,1 pce | B | E | 1,580 | 1,577 | 2,491,965 | 0.19 | 85.18 |
| 123 | Butylscopolamine 20mg/ml inj | B | V | 13,629 | 182 | 2,482,270 | 0.19 | 85.37 |
| 124 | Promethazine 5mg/ml sirop fl de 60ml | B | E | 11,460 | 204 | 2,338,227 | 0.18 | 85.55 |
| 125 | Chloramphénicol collyre à 0,5 % fl | B | E | 11,936 | 192 | 2,292,175 | 0.18 | 85.73 |
| 126 | Medical Infrared Thermometer | B | N | 38 | 59,979 | 2,279,200 | 0.17 | 85.90 |
| 127 | Valproate de sodium 200mg/ml, 100ml Syrup | B | E | 437 | 5,140 | 2,246,000 | 0.17 | 86.07 |
| 128 | Papaverine40mgcp | B | E | 110,000 | 20 | 2,227,200 | 0.17 | 86.24 |
| 129 | Ketoconazole crème 2% 15g | B | E | 8,376 | 264 | 2,215,014 | 0.17 | 86.41 |
| 130 | METFORMINE 500MG TAB B/100 | B | E | 200,900 | 11 | 2,154,130 | 0.16 | 86.58 |
| 131 | Mebendazole 100mg cp | B | E | 509,000 | 4 | 2,124,036 | 0.16 | 86.74 |
| 132 | Aminophylline 100mg cp | B | E | 611,000 | 3 | 2,116,656 | 0.16 | 86.90 |
| 133 | Hydrochlorothiazide 50mg cp | B | E | 373,000 | 6 | 2,115,242 | 0.16 | 87.07 |
| 134 | Sulfolyser 500ml sln | B | V | 18 | 115,578 | 2,080,400 | 0.16 | 87.22 |
| 135 | TOXO RAPID TEST IGG/M B/25TESTS | B | V | 76 | 27,242 | 2,070,400 | 0.16 | 87.38 |
| 136 | Catheter court IV n°20 pce | B | V | 20,192 | 102 | 2,066,158 | 0.16 | 87.54 |
| 137 | Bupivacaine Isobar 0.5% 5mg/ml fl 20ml | B | V | 533 | 3,815 | 2,033,600 | 0.16 | 87.70 |
| 138 | Whytfield ointment 50g | B | N | 2,645 | 767 | 2,027,687 | 0.16 | 87.85 |
| 139 | Albendazole 400mg Tabs B/100 | B | E | 64,700 | 31 | 1,989,005 | 0.15 | 88.00 |
| 140 | Tensiomètre digital, pce | B | V | 57 | 34,562 | 1,970,014 | 0.15 | 88.15 |
| 141 | TOXO latex 100 TEST kit | B | V | 69 | 28,007 | 1,932,500 | 0.15 | 88.30 |
| 142 | Pyridoxine 100mg cp | B | E | 137,000 | 14 | 1,928,000 | 0.15 | 88.45 |
| 143 | Aiguille dentaire 27GB 1pce | B | V | 33,105 | 57 | 1,874,866 | 0.14 | 88.59 |
| 144 | Acétylsalicylate de lysine 0.9g/5ml amp | B | V | 4,237 | 438 | 1,857,666 | 0.14 | 88.74 |
| 145 | Ciprofloxacine250mg cp | B | E | 104,000 | 17 | 1,816,319 | 0.14 | 88.87 |
| 146 | Seringue de 2ml + aiguille 23 G plastic uu pce | B | V | 65,915 | 27 | 1,800,266 | 0.14 | 89.01 |
| 147 | Aluminium Hydroxyde 500mg ces | B | E | 549,000 | 3 | 1,794,523 | 0.14 | 89.15 |
| 148 | Fentanil 0.05mg/ml amp 2ml | B | V | 660 | 2,680 | 1,768,650 | 0.14 | 89.29 |
| 149 | Hydrocortisone 100mg/ml fl inj | B | V | 6,806 | 258 | 1,755,446 | 0.13 | 89.42 |
| 150 | Lamelles Couvre objet | B | V | 293 | 5,926 | 1,736,446 | 0.13 | 89.55 |
| 151 | Thiamine 50mg | B | E | 248,000 | 7 | 1,731,644 | 0.13 | 89.68 |
| 152 | Acide nalidixique 500mg cp | B | E | 43,900 | 39 | 1,726,031 | 0.13 | 89.82 |
| 153 | Sonde vésicale de Foley 2 voies ch 16 pce | B | V | 5,142 | 331 | 1,703,822 | 0.13 | 89.95 |
| 154 | Nystatine 100 000 UI cp vaginal | B | E | 71,972 | 24 | 1,695,308 | 0.13 | 90.08 |
| 155 | Multivitamins tab | B | E | 751,000 | 2 | 1,665,473 | 0.13 | 90.20 |
| 156 | Serum antitetanique inj | B | V | 660 | 2,464 | 1,626,000 | 0.12 | 90.33 |
| 157 | ASLO KIT 100 TEST | B | E | 139 | 11,516 | 1,600,749 | 0.12 | 90.45 |
| 158 | Zinc sulfate tablet 20MG B/100 | B | E | 174,200 | 9 | 1,599,994 | 0.12 | 90.57 |
| 159 | Lidocaine 2% fl inj 30ml | B | V | 5,970 | 266 | 1,588,902 | 0.12 | 90.70 |
| 160 | Ketoconazole 200mg cp BTE B/ 1000 | B | E | 89,000 | 17 | 1,554,476 | 0.12 | 90.81 |
| 161 | Seringue a insuline 100 UI+aiguille 29G PCE | B | V | 36,600 | 41 | 1,508,820 | 0.12 | 90.93 |
| 162 | Stromatolyser 4 DS, 42ml | B | V | 13 | 113,848 | 1,480,020 | 0.11 | 91.04 |
| 163 | Diclofenac 25mg/ml inj amp de 3ml | B | V | 32,023 | 46 | 1,476,959 | 0.11 | 91.16 |
| 164 | METFORMINE 850MG TAB B/1000 | B | E | 114,400 | 13 | 1,453,155 | 0.11 | 91.27 |
| 165 | Lancette stérile UU 200 pces | B | V | 811 | 1,783 | 1,446,281 | 0.11 | 91.38 |
| 166 | Tramadol 50mg,cp | B | E | 91,800 | 16 | 1,425,749 | 0.11 | 91.49 |
| 167 | Bande plâtree 15cm x 2.7m | B | V | 4,024 | 353 | 1,421,822 | 0.11 | 91.60 |
| 168 | SOLUTION HYDROALCOOLIQUE 5L | B | E | 71 | 20,000 | 1,420,000 | 0.11 | 91.70 |
| 169 | Vitamine B Complexe cp | B | E | 938,000 | 1 | 1,405,275 | 0.11 | 91.81 |
| 170 | Test de grossesse pce | B | V | 19,560 | 70 | 1,374,864 | 0.11 | 91.92 |
| 171 | Metronidazole ovule 500mg | B | E | 17,100 | 79 | 1,353,825 | 0.10 | 92.02 |
| 172 | Glucosé 50% fl de 100ml | B | V | 1,899 | 707 | 1,342,600 | 0.10 | 92.12 |
| 173 | Cimetidine 100mg/ml 2ml inj. | B | V | 8,910 | 150 | 1,335,776 | 0.10 | 92.23 |
| 174 | Benzathine benzylpenicillin 2,4Mui | B | V | 7,218 | 183 | 1,320,318 | 0.10 | 92.33 |
| 175 | Umbilical cord clamps sterile | B | V | 20,756 | 63 | 1,305,057 | 0.10 | 92.43 |
| 176 | Set de perfusion pediatrique | B | V | 1,850 | 693 | 1,281,750 | 0.10 | 92.52 |
| 177 | AST/GOT 4+1 SL 2x6.2ML | B | V | 15 | 84,280 | 1,264,200 | 0.10 | 92.62 |
| 178 | Amitriptylline Hydro 25mg cp | B | E | 331,000 | 4 | 1,222,261 | 0.09 | 92.71 |
| 179 | Dexamethazone 0.1% collyre | B | N | 3,590 | 337 | 1,208,514 | 0.09 | 92.81 |
| 180 | RHo( D) Immune Globulin inj | B | V | 60 | 19,652 | 1,179,120 | 0.09 | 92.90 |
| 181 | Cimetidine 200 mg cp | B | E | 206,000 | 6 | 1,165,729 | 0.09 | 92.99 |
| 182 | BENZYL BENZOATE 100ML 25% | B | E | 2,304 | 506 | 1,165,600 | 0.09 | 93.08 |
| 183 | GRAM STAIN | B | V | 56 | 20,750 | 1,161,972 | 0.09 | 93.16 |
| 184 | Cinnarizine 25mg cp | B | E | 89,000 | 13 | 1,145,636 | 0.09 | 93.25 |
| 185 | Chloramphénicol 250mg gel | B | N | 61,000 | 19 | 1,130,211 | 0.09 | 93.34 |
| 186 | Sterilization Air Sec 60l | B | V | 1 | 1,119,000 | 1,119,000 | 0.09 | 93.42 |
| 187 | GLUCOSE PAP SL 6x10ML | B | V | 9 | 123,411 | 1,110,700 | 0.08 | 93.51 |
| 188 | Fer + Acide folique sp | B | E | 490 | 2,249 | 1,102,188 | 0.08 | 93.59 |
| 189 | Lame de bistouris n° 22 | B | V | 30,612 | 35 | 1,083,490 | 0.08 | 93.68 |
| 190 | Gentamicine 40mg /ml inj | B | V | 24,620 | 44 | 1,076,665 | 0.08 | 93.76 |
| 191 | HCV Ab Rapid test strip, 50 test | B | V | 100 | 10,664 | 1,066,400 | 0.08 | 93.84 |
| 192 | Chlorpheniramine 4 mg ces | B | E | 1,209,000 | 1 | 1,048,724 | 0.08 | 93.92 |
| 193 | Haloperidol 5mg cp | B | E | 166,300 | 6 | 1,044,881 | 0.08 | 94.00 |
| 194 | Tube sec vacutainer 4ml B/100 | B | V | 201 | 5,190 | 1,043,120 | 0.08 | 94.08 |
| 195 | Serum anti D (IgG IgM) Monoclonal 10ml 1 fl | B | V | 275 | 3,758 | 1,033,375 | 0.08 | 94.16 |
| 196 | RF Kit 100 Test | B | V | 106 | 9,724 | 1,030,700 | 0.08 | 94.24 |
| 197 | Chloramphenicol gouttes otique 5% | B | E | 3,620 | 268 | 970,417 | 0.07 | 94.31 |
| 198 | Giemsa soiution 0.5l | B | E | 84 | 11,310 | 950,050 | 0.07 | 94.39 |
| 199 | Furosemide 40mg cp | B | V | 310,000 | 3 | 943,042 | 0.07 | 94.46 |
| 200 | UREA UV SL 4X62.5ML | B | V | 12 | 78,085 | 937,016 | 0.07 | 94.53 |
| 201 | GLUCOSE 10% 250ML PERF | B | V | 2,195 | 426 | 934,255 | 0.07 | 94.60 |
| 202 | Dexamethazone+ Neomycine 2% Eye drop | B | E | 2,500 | 373 | 931,850 | 0.07 | 94.67 |
| 203 | Haloperidol 5mg inj | B | V | 793 | 1,162 | 921,805 | 0.07 | 94.74 |
| 204 | Amlodipine 10mg | B | E | 62,000 | 15 | 919,600 | 0.07 | 94.81 |
| 205 | Chlorpromazine 25 mg cp | B | E | 238,000 | 4 | 914,322 | 0.07 | 94.88 |
| 206 | Alcool denature 95%, 5l | B | V | 81 | 11,142 | 902,471 | 0.07 | 94.95 |
| 207 | Haemacell 30g/l fl 500ml | B | V | 113 | 7,983 | 902,100 | 0.07 | 95.02 |
| 208 | Glibenclamide 5mg cp | B | E | 337,000 | 3 | 901,465 | 0.07 | 95.09 |
| 209 | Hydrocortisone cream 20gr 1% | B | N | 3,089 | 286 | 883,795 | 0.07 | 95.16 |
| 210 | Dicynone 250 mg 1cp | C | V | 7,000 | 121 | 848,450 | 0.06 | 95.22 |
| 211 | Phenobarbital 100mg inj | C | V | 1,312 | 645 | 846,450 | 0.06 | 95.29 |
| 212 | Mosquito net white 1250x65x250 pce | C | V | 190 | 4,450 | 845,500 | 0.06 | 95.35 |
| 213 | Thermometre médical digital 1pce | C | E | 205 | 4,113 | 843,150 | 0.06 | 95.42 |
| 214 | Whitfield 20gr pde | C | N | 5,285 | 159 | 841,760 | 0.06 | 95.48 |
| 215 | Sac à Urine | C | V | 4,311 | 194 | 837,299 | 0.06 | 95.55 |
| 216 | Sonde vésicale de Foley 2 voies ch 18 pce | C | V | 2,240 | 368 | 824,602 | 0.06 | 95.61 |
| 217 | CREATININE JAFFE 2x125ML | C | V | 10 | 82,395 | 823,950 | 0.06 | 95.67 |
| 218 | Diazepam 5mg cp | C | E | 69,020 | 12 | 813,912 | 0.06 | 95.73 |
| 219 | Ephedrine 50mg/ml inj | C | V | 2,580 | 294 | 757,450 | 0.06 | 95.79 |
| 220 | Aiguille a PL 22 G 90MM | C | V | 2,536 | 298 | 756,642 | 0.06 | 95.85 |
| 221 | Pommade Camphre 100gr | C | N | 980 | 750 | 735,000 | 0.06 | 95.91 |
| 222 | Huile à Immersion | C | V | 84 | 8,749 | 734,922 | 0.06 | 95.96 |
| 223 | Comprime d'eau de Javel 150gr, 100ces | C | E | 4,805 | 151 | 723,823 | 0.06 | 96.02 |
| 224 | Promethazine 25mg cp | C | E | 206,500 | 3 | 711,719 | 0.05 | 96.07 |
| 225 | Nystatin Ointment | C | E | 365 | 1,937 | 707,133 | 0.05 | 96.13 |
| 226 | Diazepan 5mg/ml amp inj. 2ml | C | V | 1,790 | 394 | 705,875 | 0.05 | 96.18 |
| 227 | Serum anti-B Monoclonal 10ml 1 flacon Biotec | C | V | 274 | 2,564 | 702,412 | 0.05 | 96.23 |
| 228 | Glass ionomer(GC Gold label) kit | C | E | 10 | 66,050 | 660,500 | 0.05 | 96.28 |
| 229 | ALT/GPT 4+1SL 2x62.ML | C | V | 12 | 55,000 | 660,000 | 0.05 | 96.33 |
| 230 | Serum anti A Monoclonal 10ml 1 flacon Biotec | C | V | 263 | 2,503 | 658,376 | 0.05 | 96.38 |
| 231 | Prednisolone 1% eye drop | C | E | 580 | 1,118 | 648,300 | 0.05 | 96.43 |
| 232 | Human GPT KIT | C | V | 18 | 35,540 | 639,720 | 0.05 | 96.48 |
| 233 | Fil NR Synth 75cm PR Nº1 1/2C 40mm B/12 | C | V | 268 | 2,348 | 629,351 | 0.05 | 96.53 |
| 234 | Pommade ichytiol 10% pot de 50g | C | N | 463 | 1,342 | 621,443 | 0.05 | 96.58 |
| 235 | URIC ACID MONO SL 6x50ML | C | V | 5 | 123,300 | 616,500 | 0.05 | 96.63 |
| 236 | Chloramphénicol inj 1g | C | V | 2,216 | 277 | 614,763 | 0.05 | 96.67 |
| 237 | HC Eolyse 1L | C | E | 6 | 101,167 | 607,000 | 0.05 | 96.72 |
| 238 | Paracétamol 100mg cp | C | E | 422,000 | 1 | 600,015 | 0.05 | 96.77 |
| 239 | Comprime d'eau de javel 150g, 48pastilles | C | E | 95 | 6,297 | 598,200 | 0.05 | 96.81 |
| 240 | REVELATEUR FILM RADIO 20 LTRS | C | E | 12 | 48,146 | 577,750 | 0.04 | 96.86 |
| 241 | Magnesium trisilicate 550mg cp | C | E | 260,000 | 2 | 575,385 | 0.04 | 96.90 |
| 242 | Paracetamol 100mg/ml inj | C | V | 550 | 1,036 | 570,000 | 0.04 | 96.94 |
| 243 | Abaisse langue en bois pce | C | E | 13,809 | 41 | 568,122 | 0.04 | 96.99 |
| 244 | Acétylsalicylique acide 100mg cp | C | E | 192,000 | 3 | 554,902 | 0.04 | 97.03 |
| 245 | Film Radiologie 35cm x 43cm, pce | C | V | 600 | 910 | 546,200 | 0.04 | 97.07 |
| 246 | RPR KIT 100 TEST | C | V | 79 | 6,848 | 541,000 | 0.04 | 97.11 |
| 247 | Violet de gentiane poudre 25g | C | V | 76 | 7,074 | 537,600 | 0.04 | 97.15 |
| 248 | Biperdene 2 MG TAB | C | E | 8,250 | 65 | 532,275 | 0.04 | 97.19 |
| 249 | Promethazine 100ml sp | C | E | 2,015 | 263 | 529,990 | 0.04 | 97.23 |
| 250 | Serum anti-AB Monoclonal 10ml 1 flacon Biot | C | V | 167 | 3,093 | 516,511 | 0.04 | 97.27 |
| 251 | Magnesium sulfate 50% 10ml inj | C | V | 879 | 586 | 515,100 | 0.04 | 97.31 |
| 252 | Salbutamol 4mg cp | C | E | 380,000 | 1 | 502,583 | 0.04 | 97.35 |
| 253 | Compresse paraffine 10x10cm(Tulle gras) | C | V | 4,840 | 103 | 499,570 | 0.04 | 97.39 |
| 254 | EE SYSTEM SOLUTION 1 L | C | E | 6 | 83,100 | 498,600 | 0.04 | 97.43 |
| 255 | Clotrimazole 100mg, cp vaginal | C | E | 10,182 | 48 | 485,084 | 0.04 | 97.47 |
| 256 | ELITROL II 10x5ML | C | E | 5 | 95,194 | 475,970 | 0.04 | 97.50 |
| 257 | Morphine 10mg/ml, inj | C | V | 980 | 484 | 474,608 | 0.04 | 97.54 |
| 258 | Cotrimoxazole à 120mg cp | C | E | 122,000 | 4 | 469,537 | 0.04 | 97.57 |
| 259 | Furosemide 10mg/ml 2ml inj | C | V | 8,250 | 56 | 462,072 | 0.04 | 97.61 |
| 260 | Aminophylline 25mg/ml amp de 10ml | C | V | 4,670 | 98 | 459,785 | 0.04 | 97.64 |
| 261 | Sparadrap perforé 18cmx5m pce | C | V | 190 | 2,329 | 442,500 | 0.03 | 97.68 |
| 262 | ISE CONTROL I 10X5ML | C | E | 3 | 145,600 | 436,800 | 0.03 | 97.71 |
| 263 | Permanganate de potassium 500mg ce | C | N | 8,550 | 51 | 435,861 | 0.03 | 97.75 |
| 264 | Glucose 10% 500ml | C | V | 660 | 656 | 433,200 | 0.03 | 97.78 |
| 265 | Nitrofurantoine 100 MG CP | C | E | 77,000 | 6 | 429,508 | 0.03 | 97.81 |
| 266 | Aniosgel 500ml | C | E | 50 | 8,500 | 425,000 | 0.03 | 97.84 |
| 267 | Chlorpromazine 50mg/2ml inj (Largactil) | C | V | 2,480 | 171 | 423,687 | 0.03 | 97.88 |
| 268 | HC BASOLYSE | C | E | 4 | 105,250 | 421,000 | 0.03 | 97.91 |
| 269 | Gel pour echographie 5 L | C | V | 40 | 10,404 | 416,140 | 0.03 | 97.94 |
| 270 | Centrifugeuse electriquen220V | C | E | 1 | 416,000 | 416,000 | 0.03 | 97.97 |
| 271 | ELICAL 2-4X3ML | C | E | 4 | 102,533 | 410,130 | 0.03 | 98.00 |
| 272 | Haloperidol Decanoate inj | C | V | 67 | 6,075 | 406,996 | 0.03 | 98.03 |
| 273 | CHOLESTEROL 6X100ML | C | E | 1 | 406,760 | 406,760 | 0.03 | 98.07 |
| 274 | ELITROL I 10x5ML | C | E | 4 | 101,663 | 406,650 | 0.03 | 98.10 |
| 275 | Morphine 10mg Tab | C | E | 1,440 | 280 | 403,200 | 0.03 | 98.13 |
| 276 | Pommade antihemorroide pot de 30g | C | E | 350 | 1,102 | 385,720 | 0.03 | 98.16 |
| 277 | Cinnarizine 75 mg cp | C | E | 14,000 | 27 | 381,450 | 0.03 | 98.19 |
| 278 | Amalgame dentaire capsule | C | E | 7 | 54,486 | 381,400 | 0.03 | 98.22 |
| 279 | Sonde d'aspiration CH 8 | C | V | 2,584 | 147 | 380,390 | 0.03 | 98.25 |
| 280 | Glucometer CODE free | C | V | 39 | 9,667 | 377,000 | 0.03 | 98.27 |
| 281 | Fil 35 x 43 | C | E | 300 | 1,250 | 375,000 | 0.03 | 98.30 |
| 282 | Gauze Abdominal 45cmx45cm PCE | C | V | 800 | 462 | 369,840 | 0.03 | 98.33 |
| 283 | Gentamycine 0.3% 10ml collyre | C | E | 2,283 | 161 | 367,443 | 0.03 | 98.36 |
| 284 | Human GOT KIT | C | V | 11 | 32,500 | 357,500 | 0.03 | 98.39 |
| 285 | ACID SOLUTION 1 L | C | V | 4 | 88,721 | 354,882 | 0.03 | 98.41 |
| 286 | Aiguille Poncti Lomb Luer 22G 90mm(spinall | C | V | 1,000 | 345 | 344,981 | 0.03 | 98.44 |
| 287 | Ketamine 50MG/ML, FL 10ML inj | C | V | 214 | 1,608 | 344,040 | 0.03 | 98.47 |
| 288 | Hydrocortisone eye drop 5ml 1 % | C | E | 780 | 438 | 341,710 | 0.03 | 98.49 |
| 289 | Alcohol 70%, 1 litter | C | V | 40 | 8,511 | 340,433 | 0.03 | 98.52 |
| 290 | Lactulose (Duphalac sol) 3.33gr/5ml | C | E | 180 | 1,883 | 339,000 | 0.03 | 98.54 |
| 291 | Atenolol 100mg | C | E | 27,600 | 12 | 335,061 | 0.03 | 98.57 |
| 292 | Polyglactin no. 2/0 910 75cm B/12 | C | V | 36 | 9,300 | 334,800 | 0.03 | 98.60 |
| 293 | Pénicilline Procaine 1mui+3mui fl | C | V | 1,705 | 195 | 332,737 | 0.03 | 98.62 |
| 294 | Halothane inhalation fl 250ml | C | V | 10 | 33,064 | 330,642 | 0.03 | 98.65 |
| 295 | Prednisolone 0.5% eye drop | C | E | 240 | 1,351 | 324,210 | 0.02 | 98.67 |
| 296 | HC Cleaner 1L | C | V | 5 | 64,800 | 324,000 | 0.02 | 98.70 |
| 297 | Clomipramine 25mg cp (anafranil 25mg) cp | C | E | 10,360 | 31 | 320,428 | 0.02 | 98.72 |
| 298 | Tramadol hydrochloride 100mg/2ml, inj | C | V | 1,470 | 214 | 314,148 | 0.02 | 98.74 |
| 299 | Ibuprofen sirop 60ml fl | C | E | 1,160 | 270 | 313,200 | 0.02 | 98.77 |
| 300 | Tube vacutainer EDTA 4ML B/100 | C | V | 65 | 4,793 | 311,550 | 0.02 | 98.79 |
| 301 | Catheter Court IV, UU G22 | C | V | 2,670 | 116 | 309,766 | 0.02 | 98.82 |
| 302 | Fil 30x 40 | C | V | 400 | 735 | 293,808 | 0.02 | 98.84 |
| 303 | EAU OXYGENEE 120ML | C | E | 1,105 | 264 | 291,685 | 0.02 | 98.86 |
| 304 | ISE CONTROL II 10X5ML | C | E | 2 | 145,600 | 291,200 | 0.02 | 98.88 |
| 305 | Fixateur Film Solution Concentree RX 20L | C | E | 15 | 19,408 | 291,115 | 0.02 | 98.91 |
| 306 | Film Radiologie 30 cmx 40cm,pce | C | E | 400 | 727 | 290,943 | 0.02 | 98.93 |
| 307 | SURFANIOS PREMIUM 1L | C | N | 10 | 28,000 | 280,000 | 0.02 | 98.95 |
| 308 | Salbutamol sirop 100ml | C | E | 1,042 | 263 | 274,010 | 0.02 | 98.97 |
| 309 | Micropipette 10-100ml, 1pce | C | V | 2 | 136,750 | 273,500 | 0.02 | 98.99 |
| 310 | ISE CALIBRATOR(6x20ML+6X20ML) | C | E | 2 | 135,000 | 270,000 | 0.02 | 99.01 |
| 311 | Griseofulvine 125 mg cp | C | E | 11,500 | 23 | 267,130 | 0.02 | 99.03 |
| 312 | Miconazole crème | C | N | 1,666 | 160 | 267,060 | 0.02 | 99.05 |
| 313 | Dexamethasone Tabs 0.5mg B/1000 | C | E | 61,000 | 4 | 265,840 | 0.02 | 99.07 |
| 314 | Papaverine 40mg/2ml inj | C | V | 904 | 293 | 264,478 | 0.02 | 99.09 |
| 315 | Phenobarbital 50mg,cp | C | E | 37,000 | 7 | 259,000 | 0.02 | 99.11 |
| 316 | Oxytocine inj.10UI, IM | C | V | 1,010 | 253 | 255,736 | 0.02 | 99.13 |
| 317 | Quinine 300mg cp | C | E | 30,100 | 8 | 253,021 | 0.02 | 99.15 |
| 318 | ISE REFERENCE SOLUTION 1x500ML | C | E | 4 | 62,540 | 250,160 | 0.02 | 99.17 |
| 319 | TAMBOUR DE STERILISATION 190x150, 1 | C | V | 5 | 50,000 | 250,000 | 0.02 | 99.19 |
| 320 | Dexamethasone 4mg/2ml inj | C | V | 6,070 | 41 | 247,696 | 0.02 | 99.21 |
| 321 | Acyclovir 200mg cp | C | E | 12,580 | 19 | 244,112 | 0.02 | 99.23 |
| 322 | Bisacodyl 5mg cp | C | N | 35,200 | 7 | 231,998 | 0.02 | 99.25 |
| 323 | Sparadrap tissé 2cm*5m pce | C | V | 579 | 395 | 228,705 | 0.02 | 99.26 |
| 324 | Pethidine 50mg/ml 1ml amp inj | C | V | 430 | 527 | 226,479 | 0.02 | 99.28 |
| 325 | THERMOMETRE MURAL 1 PCE | C | E | 15 | 15,000 | 225,000 | 0.02 | 99.30 |
| 326 | Hydralazine 20mg inj | C | V | 85 | 2,563 | 217,820 | 0.02 | 99.31 |
| 327 | Eau Oxygenee 200ml | C | E | 87 | 2,414 | 210,018 | 0.02 | 99.33 |
| 328 | Albendazole Syrup 100mg/5ml 20ml | C | E | 1,294 | 153 | 197,728 | 0.02 | 99.35 |
| 329 | Film digital x-ray 25cmX30cm | C | E | 176 | 1,093 | 192,280 | 0.01 | 99.36 |
| 330 | Bassin reniforme en inox | C | E | 10 | 19,200 | 192,000 | 0.01 | 99.37 |
| 331 | HC Lyse 1L | C | V | 2 | 90,000 | 180,000 | 0.01 | 99.39 |
| 332 | Javel solution 5L | C | V | 12 | 15,000 | 180,000 | 0.01 | 99.40 |
| 333 | Lunette nasale pr Adult | C | V | 235 | 737 | 173,232 | 0.01 | 99.42 |
| 334 | Sonde Gastrique LEVIN UU CH 5 | C | V | 1,318 | 131 | 172,490 | 0.01 | 99.43 |
| 335 | Lunette nasale pr Pediatrique (Child) | C | V | 260 | 653 | 169,770 | 0.01 | 99.44 |
| 336 | HYDROALCOHOLIC GEL 300ml | C | E | 36 | 4,677 | 168,372 | 0.01 | 99.45 |
| 337 | Phenytoine 100 mg tab | C | E | 26,000 | 6 | 167,785 | 0.01 | 99.47 |
| 338 | Bande platrée 10cm x 2.7m pce | C | V | 628 | 258 | 162,130 | 0.01 | 99.48 |
| 339 | ISE DILUENT 12x25ML | C | E | 2 | 79,690 | 159,380 | 0.01 | 99.49 |
| 340 | GAMMA GT PLUS SL 8X25ML | C | V | 2 | 79,500 | 159,000 | 0.01 | 99.50 |
| 341 | Metoclopramide 5mg/ml amp 2ml | C | V | 2,925 | 54 | 158,760 | 0.01 | 99.52 |
| 342 | Bande Crepe 10 cm* 4.5m pce | C | E | 694 | 228 | 158,380 | 0.01 | 99.53 |
| 343 | Violet de gentiane 2% fl de 1l | C | E | 14 | 10,986 | 153,800 | 0.01 | 99.54 |
| 344 | EE SYSTEM CLEANING SOLUTION 1 L | C | E | 1 | 153,600 | 153,600 | 0.01 | 99.55 |
| 345 | Cidezyme (Aniosyme) 5l | C | N | 3 | 50,390 | 151,170 | 0.01 | 99.56 |
| 346 | Métoclopramide à 10mg cp | C | E | 48,000 | 3 | 149,096 | 0.01 | 99.57 |
| 347 | Pyridoxine 50mg tab | C | E | 17,300 | 9 | 148,340 | 0.01 | 99.59 |
| 348 | Gel pour Echographie 250ml | C | V | 210 | 672 | 141,200 | 0.01 | 99.60 |
| 349 | Promethazine 25mg /ml inj 2ml amp | C | V | 1,160 | 121 | 140,258 | 0.01 | 99.61 |
| 350 | Atropine 1% collyre | C | E | 115 | 1,189 | 136,750 | 0.01 | 99.62 |
| 351 | Sulfadiazine 20gr cream | C | E | 450 | 297 | 133,600 | 0.01 | 99.63 |
| 352 | AMYLASE 6x20ml | C | E | 1 | 126,000 | 126,000 | 0.01 | 99.64 |
| 353 | Charbon activé 125mg cp | C | E | 2,300 | 54 | 125,081 | 0.01 | 99.65 |
| 354 | Aniosgel 1L | C | E | 9 | 13,500 | 121,500 | 0.01 | 99.66 |
| 355 | CLEANER+CONDITION 6X8ML | C | E | 1 | 117,450 | 117,450 | 0.01 | 99.67 |
| 356 | Adrenaline 1mg/ml, 1ml inj | C | V | 1,590 | 71 | 112,252 | 0.01 | 99.67 |
| 357 | Sonde d'aspiration CH 10 | C | V | 785 | 141 | 110,942 | 0.01 | 99.68 |
| 358 | Catgut chrome dec 5(1),75cm PR 1/2C, 40m | C | V | 28 | 3,888 | 108,850 | 0.01 | 99.69 |
| 359 | Atenolol 50mg tab | C | E | 10,900 | 10 | 104,420 | 0.01 | 99.70 |
| 360 | Pince de kocher droite 16cm | C | E | 20 | 5,200 | 104,000 | 0.01 | 99.71 |
| 361 | Oxyde de zinc 50gr pde | C | N | 163 | 613 | 99,940 | 0.01 | 99.72 |
| 362 | Boite a Instrument 20x10x50cm | C | E | 5 | 19,630 | 98,150 | 0.01 | 99.72 |
| 363 | Pince hemostatique 16cm | C | E | 20 | 4,775 | 95,500 | 0.01 | 99.73 |
| 364 | Bilirubin D | C | E | 1 | 90,450 | 90,450 | 0.01 | 99.74 |
| 365 | Bilurbin T | C | E | 1 | 90,450 | 90,450 | 0.01 | 99.74 |
| 366 | Dopamine 200mg Inj | C | V | 40 | 2,250 | 90,000 | 0.01 | 99.75 |
| 367 | Glycerine 2.5L | C | N | 3 | 29,833 | 89,500 | 0.01 | 99.76 |
| 368 | Phenobarbutal 30mg cp | C | E | 13,000 | 7 | 84,500 | 0.01 | 99.76 |
| 369 | Mannitol 20% 100ml fl | C | V | 56 | 1,504 | 84,200 | 0.01 | 99.77 |
| 370 | Pissette en plastique 250ml | C | E | 21 | 4,005 | 84,100 | 0.01 | 99.78 |
| 371 | Spectinomycin 2g Powder Inject | C | V | 27 | 3,019 | 81,500 | 0.01 | 99.78 |
| 372 | Bicarbonate de Sodium pdre 50gr | C | N | 294 | 273 | 80,374 | 0.01 | 99.79 |
| 373 | Ciseau droit a bout mousse 16cm | C | E | 10 | 7,800 | 78,000 | 0.01 | 99.80 |
| 374 | Dicynone 250mg/2ml inj | C | V | 128 | 600 | 76,800 | 0.01 | 99.80 |
| 375 | Warfarin 5mg tab | C | E | 1,500 | 50 | 75,645 | 0.01 | 99.81 |
| 376 | Aiguille hypodermique,21G pce | C | V | 6,100 | 12 | 74,490 | 0.01 | 99.81 |
| 377 | Lames de bistouri No 23 | C | V | 2,100 | 34 | 71,715 | 0.01 | 99.82 |
| 378 | TOTAL PROTEIN PLUS 12X20ML | C | E | 1 | 70,200 | 70,200 | 0.01 | 99.82 |
| 379 | Bupivacaine hyperbale 0.5mg/ml amp 4ml inj | C | V | 100 | 700 | 70,000 | 0.01 | 99.83 |
| 380 | Tube centrif.Plastic Gradueel, 1 pce | C | E | 100 | 700 | 70,000 | 0.01 | 99.83 |
| 381 | Stéthoscope médical | C | E | 14 | 4,896 | 68,545 | 0.01 | 99.84 |
| 382 | Pince Porte aiguille de Mayor-Hegar 16 cm | C | E | 10 | 6,515 | 65,150 | 0.00 | 99.84 |
| 383 | Pince Mosquito 12cm | C | E | 10 | 6,320 | 63,200 | 0.00 | 99.85 |
| 384 | Atenolol 25mg tab | C | E | 2,850 | 21 | 60,990 | 0.00 | 99.85 |
| 385 | Bandage Jersey tub 10cmX 25cm | C | E | 3 | 18,241 | 54,722 | 0.00 | 99.86 |
| 386 | Acide folique 5mg cp | C | E | 23,000 | 2 | 53,088 | 0.00 | 99.86 |
| 387 | SPECTINOMYCINE 2G POUDRE +SOLVANT | C | E | 15 | 3,500 | 52,500 | 0.00 | 99.87 |
| 388 | Timolol 0.5% 5ML collyre | C | N | 60 | 838 | 50,250 | 0.00 | 99.87 |
| 389 | Ciseau courbe à bout mousse 16cm, 1 pce | C | E | 10 | 5,000 | 50,000 | 0.00 | 99.87 |
| 390 | Ciseau courbe à bout Mousse 18cm, 1 pce | C | E | 10 | 5,000 | 50,000 | 0.00 | 99.88 |
| 391 | Aniosgel 5L | C | E | 1 | 49,500 | 49,500 | 0.00 | 99.88 |
| 392 | Ciseau droit à bout Mousse 14cm | C | E | 10 | 4,700 | 47,000 | 0.00 | 99.88 |
| 393 | Syringe 50ml | C | E | 200 | 230 | 46,000 | 0.00 | 99.89 |
| 394 | Urea human 200ml Kit | C | E | 2 | 23,000 | 46,000 | 0.00 | 99.89 |
| 395 | Vitamine B Complex inj | C | V | 550 | 81 | 44,400 | 0.00 | 99.90 |
| 396 | Ecouvillons non steriles, bois,coton | C | E | 135 | 323 | 43,540 | 0.00 | 99.90 |
| 397 | Sonde Gastrique LEVIN UU CH 8 | C | V | 325 | 132 | 42,875 | 0.00 | 99.90 |
| 398 | Suxamethonium 50mg/2ml inj | C | V | 35 | 1,219 | 42,650 | 0.00 | 99.91 |
| 399 | Sonde foley 2 voies sterile uu ch 14, pc | C | E | 150 | 283 | 42,450 | 0.00 | 99.91 |
| 400 | Vaseline Blanche 250gr | C | E | 44 | 953 | 41,950 | 0.00 | 99.91 |
| 401 | Prednisolone 10mg ce | C | E | 3,000 | 14 | 41,400 | 0.00 | 99.91 |
| 402 | Neostigmine inj 0.5mg | C | V | 40 | 1,026 | 41,050 | 0.00 | 99.92 |
| 403 | Atropine sulfate 0.5mg/ml inj | C | V | 488 | 81 | 39,635 | 0.00 | 99.92 |
| 404 | Glycerine pure 1L | C | N | 4 | 9,900 | 39,600 | 0.00 | 99.92 |
| 405 | Fil 18x 24 | C | E | 100 | 386 | 38,612 | 0.00 | 99.93 |
| 406 | Eau oxygenee 250ml | C | E | 96 | 400 | 38,400 | 0.00 | 99.93 |
| 407 | Phenytoin sodium 250mg/5ml inj | C | V | 40 | 960 | 38,400 | 0.00 | 99.93 |
| 408 | Violet de gentiane 500ml | C | E | 8 | 4,800 | 38,400 | 0.00 | 99.94 |
| 409 | Aiguilles à PL UU G25 | C | V | 125 | 304 | 38,050 | 0.00 | 99.94 |
| 410 | Film Radiologie 18cm x 24cm, pce | C | E | 100 | 355 | 35,453 | 0.00 | 99.94 |
| 411 | Pissette en Plastique 500ml | C | E | 10 | 3,500 | 35,000 | 0.00 | 99.94 |
| 412 | Sonde vesicale de foley à 2 voies ch12 pce | C | V | 100 | 340 | 34,000 | 0.00 | 99.95 |
| 413 | Bupivacaine isobare 0.5mg/ml amp 4ml | C | V | 65 | 500 | 32,500 | 0.00 | 99.95 |
| 414 | Otoscope medical | C | E | 2 | 16,100 | 32,200 | 0.00 | 99.95 |
| 415 | Sonde Gastrique LEVIN UU CH 16 | C | V | 207 | 153 | 31,581 | 0.00 | 99.95 |
| 416 | Sonde GAstrique levin UU CH 6 PC | C | V | 119 | 257 | 30,564 | 0.00 | 99.96 |
| 417 | Alcohol Acetone 1l | C | E | 2 | 14,000 | 28,000 | 0.00 | 99.96 |
| 418 | Norfloxacine 400mg cp | C | E | 1,000 | 27 | 27,169 | 0.00 | 99.96 |
| 419 | Catheter court IV n°16 pce | C | V | 200 | 135 | 27,000 | 0.00 | 99.96 |
| 420 | Nifedipine 10mg cp | C | E | 5,220 | 5 | 25,990 | 0.00 | 99.96 |
| 421 | Syringes 60ml pce | C | V | 110 | 228 | 25,060 | 0.00 | 99.97 |
| 422 | Developer Film sol 20Ls | C | E | 1 | 24,000 | 24,000 | 0.00 | 99.97 |
| 423 | Lunette nasale (Infant) neonatale | C | V | 25 | 900 | 22,500 | 0.00 | 99.97 |
| 424 | Film radio dentaire 3.1cm x 4.1cm, 150pc | C | E | 1 | 22,369 | 22,369 | 0.00 | 99.97 |
| 425 | Sonde gastrique CH18 | C | V | 140 | 160 | 22,367 | 0.00 | 99.97 |
| 426 | Aiguille hypodermique,23G | C | V | 2,000 | 11 | 21,000 | 0.00 | 99.98 |
| 427 | Gentamycine80mg/2ml 1 amp | C | V | 700 | 30 | 20,800 | 0.00 | 99.98 |
| 428 | BUPIVACAINE ISOBAR 0.5% 5MG/ML 10ML | C | V | 50 | 400 | 19,980 | 0.00 | 99.98 |
| 429 | Warfarin 2mg tab | C | E | 500 | 38 | 19,105 | 0.00 | 99.98 |
| 430 | Lidocaine 2% fl inj 20ml | C | V | 50 | 367 | 18,367 | 0.00 | 99.98 |
| 431 | Propranolol chlorhydrate 40mg cp | C | E | 4,800 | 4 | 17,049 | 0.00 | 99.98 |
| 432 | Quinine 300mg/Ml Injection B/100 | C | V | 505 | 31 | 15,655 | 0.00 | 99.98 |
| 433 | Autoclave Tape pce | C | E | 4 | 3,900 | 15,600 | 0.00 | 99.98 |
| 434 | Insulin Mixte 30/70 | C | V | 5 | 3,050 | 15,250 | 0.00 | 99.99 |
| 435 | Collecteur pour aiguilles | C | E | 14 | 1,083 | 15,167 | 0.00 | 99.99 |
| 436 | Ferroplex sirop 100ml | C | E | 50 | 300 | 15,000 | 0.00 | 99.99 |
| 437 | BANDE PLATRE 20X2.7 | C | E | 30 | 473 | 14,190 | 0.00 | 99.99 |
| 438 | Lame de bistouri n°20 | C | V | 601 | 23 | 13,823 | 0.00 | 99.99 |
| 439 | Seringue 20ml pce | C | V | 170 | 81 | 13,817 | 0.00 | 99.99 |
| 440 | Chlorure de potassium (KCl) inj. | C | V | 20 | 650 | 13,000 | 0.00 | 99.99 |
| 441 | Clotrimazole 1% crème, 20g | C | N | 60 | 195 | 11,700 | 0.00 | 99.99 |
| 442 | Violet de gentiane usage ext.sol.FL 0.5l | C | E | 2 | 4,800 | 9,600 | 0.00 | 99.99 |
| 443 | Digoxin 0.250mg,cp | C | E | 1,000 | 9 | 8,585 | 0.00 | 99.99 |
| 444 | Thermomètre médical pce | C | E | 17 | 500 | 8,500 | 0.00 | 100.00 |
| 445 | Glycerine anhydre 1L | C | N | 2 | 3,800 | 7,600 | 0.00 | 100.00 |
| 446 | Sonde gastrique CH10 | C | V | 50 | 138 | 6,900 | 0.00 | 100.00 |
| 447 | Alcohol Methylated 1 L | C | E | 2 | 3,417 | 6,834 | 0.00 | 100.00 |
| 448 | Tube d'intubation No.6.5 | C | V | 9 | 660 | 5,940 | 0.00 | 100.00 |
| 449 | Sonde d'aspiration uu ch 14 | C | V | 30 | 180 | 5,407 | 0.00 | 100.00 |
| 450 | Naloxone 0.4MG/ML Amp inj | C | V | 8 | 667 | 5,333 | 0.00 | 100.00 |
| 451 | Gel Ecographique 1L | C | V | 2 | 2,000 | 4,000 | 0.00 | 100.00 |
| 452 | Endotracheal tube 6mm+pilot balloon | C | V | 4 | 966 | 3,864 | 0.00 | 100.00 |
| 453 | Sonde d'aspiration CH 16 pce | C | V | 21 | 180 | 3,780 | 0.00 | 100.00 |
| 454 | FORMOL 37% 1L | C | E | 1 | 2,500 | 2,500 | 0.00 | 100.00 |
| 455 | Sonde d'aspiration UU CH 12 pc | C | V | 20 | 125 | 2,500 | 0.00 | 100.00 |
| 456 | EAU OXYGENE Peroxyde d'hydrogène à 3% | C | E | 7 | 280 | 1,960 | 0.00 | 100.00 |
| 457 | Clotrimazole 1% 20gr creme | C | N | 10 | 150 | 1,500 | 0.00 | 100.00 |
| **TOTAL** | | | | | | **1,306,944,142** | **100.00** |  |
