# Supplementary material for: Analysis of pharmaceutical inventory management based on ABC-VEN analysis in Rwanda: a case study of Nyamagabe district
Source: J Pharm Policy Pract. 2023 Feb 24;16:30. doi: 10.1186/s40545-023-00540-5 (PMC10129016; doi:10.1186/s40545-023-00540-5)
Supplement: Supplementary file 2 — Additional file 2: Table S2. ABC-VEN analysis in subcategories. [file 40545_2023_540_MOESM2_ESM.docx]

**S2 Table: ABC - VEN Analysis in subcategories**

| **VEN Classification subcategories** | **Number of items** | **Percentage** | **Amount**  **Rwf** | **Percentage of the value** |
| --- | --- | --- | --- | --- |
| AV | 37 | 8.09 | 366,235,331 | 28.02 |
| AE | 52 | 11.37 | 566,263,702 | 48.38 |
| AN | 1 | 0.22 | 16,956,785 | 1.30 |
| BV | 57 | 12.47 | 102,609,62 | 7.85 |
| BE | 55 | 12.05 | 111,988,625 | 8.57 |
| BN | 7 | 1.53 | 13,534,829 | 1.04 |
| CV | 108 | 23.63 | 30,351,798 | 2.32 |
| CE | 124 | 27.11 | 28,989,129 | 2.22 |
| CN | 16 | 3.50 | 3,944,756 | 0.30 |
